# Somatic mtDNA variation is an important component of Parkinson's disease

**DOI:** 10.1016/j.neurobiolaging.2015.10.036

**Published:** 2016-02

**Authors:** Jonathan Coxhead, Marzena Kurzawa-Akanbi, Rafiqul Hussain, Angela Pyle, Patrick Chinnery, Gavin Hudson

**Affiliations:** Mitochondrial Research Group, Institute of Genetic Medicine, University of Newcastle Upon Tyne, UK

**Keywords:** Mitochondria, Parkinson's disease, Neurodegeneration, Somatic mutation

## Abstract

There is a growing body of evidence linking mitochondrial dysfunction, mediated either through inherited mitochondrial DNA (mtDNA) variation or mitochondrial proteomic deficit, to Parkinson's disease (PD). Yet, despite this, the role of somatic mtDNA point mutations and specifically point-mutational burden in PD is poorly understood. Here, we take advantage of recent technical and methodological advances to examine the role of age-related and acquired mtDNA mutation in the largest study of mtDNA in postmortem PD tissue to date. Our data show that PD patients suffer an increase in mtDNA mutational burden in, but no limited to, the substantia nigra pars compacta when compared to matched controls. This mutational burden appears increased in genes encoding cytochrome c oxidase, supportive of previous protein studies of mitochondrial dysfunction in PD. Accepting experimental limitations, our study confirms the important role of age-related mtDNA point mutation in the etiology of PD, moreover, by analyzing 2 distinct brain regions, we are able to show that PD patient brains are more vulnerable to mtDNA mutation overall.

## 1 Introduction

Mitochondria are critical subcellular organelles, charged with providing cellular energy (adenosine triphosphate [ATP]) through oxidative phosphorylation (OXPHOS) by the respiratory chain. Thirteen of the ∼90 OXPHOS proteins are encoded in by mitochondrial DNA (mtDNA); a highly mutable, maternally inherited, DNA molecule, which undergoes negligible intermolecular recombination. The breakdown of OXPHOS, a disruption of cellular energy supply and demand, often leads to disease and is mediated largely through a purported vicious cycle of reactive oxygen species dependent mutation formation (de Grey, 2005).

Parkinson's disease (PD) is the second most common neurodegenerative disorder after Alzheimer's disease, affecting approximately 1% of the worldwide elderly population. Pathologically, the disease is well-characterized as a progressive loss degeneration of the substantia nigra pars compacta (SNpc), yet, despite extensive study the causes of PD remain elusive; with <10% of cases caused by a genetic defect. Multiple lines of evidence implicate mitochondrial dysfunction in the pathogenesis of idiopathic PD, with genetic analysis focusing largely on the role of extant, inherited, mtDNA variants (Ghezzi et al., 2005, Hudson et al., 2013, Hudson et al., 2014, Latsoudis et al., 2008). Reports have indicated that isolated mtDNA deletion formation is important (Bender et al., 2006, Kraytsberg et al., 2006), but the role of somatic single nucleotide variation has never been fully investigated in PD.

To assess the role of single nucleotide variants in PD, we used next generation sequencing (NGS) to analyze the mtDNA from the SNpc and frontal cortex (FC) of 180 idiopathic PD cases, comparing mtDNA mutational burden to age-matched asymptomatic control tissues.

## 2 Materials and methods

### 2.1 Cohort

Tissue samples, FC (n = 220) and mid-brain (n = 190), were obtained from the Newcastle Brain Tissue Resource (NBTR, Newcastle upon Tyne, UK), the Parkinson's UK Brain Bank (Imperial College London, London, UK), and the MRC Edinburgh Brain and Tissue Bank (University of Edinburgh, Edinburgh, UK). Patient samples (n = 180) were all Caucasian community-based PD cases, fulfilling UK-PD Society brain bank criteria for the diagnosis of PD (M = 106 and F = 74, average age at death = 78.0 years; Hughes et al., 1992). Control samples (n = 40) were all Braak stage <II (Braak et al., 2003), showing no signs of clinical or neuropathological PD (M = 22 and F = 18, average age = 77.9 years). Mean postmortem delay was 14.2 hours (Standard deviation [StDev] = 12.2 hours) in cases and 11.6 hours in controls (StDev = 13.4 hours), with no significant differences between brain banks.

### 2.2 MtDNA isolation and enrichment

SNpc was micro-dissected at 4 °C from whole mid-brain sections (10 × 20 μm sections per sample; Hudson et al., 2011), FC samples were extracted from 50–100 mg tissue blocks. To maximize DNA yield from limited tissue, total DNA was extracted from 190 SN samples (150 cases and 40 controls) and 220 FC samples (180 cases and 40 controls) using Qiagen DNA micro kit (Qiagen, Hilden, Germany).

MtDNA was enriched using long-range polymerase chain reaction. To eliminate the potential for error and nuclear DNA contamination, amplicons were polymerized using PrimeSTAR GXL DNA polymerase (error rate = 0.00108%, Takara Bio, Saint-Germain-en-Laye, France) in 2 overlapping fragments, using primer set-1: CCC TCT CTC CTA CTC CTG-F (m.6222–6239) and CAG GTG GTC AAG TAT TTA TGG–R (m.16133–16,153), and set-2: CAT CTT GCC CTT CAT TAT TGC–F (m.15295–15,315) and GGC AGG ATA GTT CAG ACG-R (7773–7791). To avoid run-specific amplification variation, case and control SN and FC samples were randomly mixed in each isolation and enrichment experiment. Initially, primer efficiency and specificity was assessed as successful after zero amplification of DNA from Rho0 cell lines, avoiding the unintended amplification of nuclear pseudogenes.

Amplified products were assessed by gel electrophoresis, against DNA+ve and DNA−ve controls, and quantified using a Qubit 2.0 fluorimeter (Life Technologies, Paisley, UK). Each amplicon was individually purified using Agencourt AMPure XP beads (Beckman-Coulter, USA), pooled in equimolar concentrations and requantified. Successful long-range polymerase chain reaction amplification, based on quality and final concentration, was successful in 174 SN (92%) and 209 FC (95%) samples.

### 2.3 MtDNA sequencing

Pooled amplicons were “tagmented,” amplified, cleaned, normalized, and pooled into 48 sample multiplexes using the Illumina Nextera XT DNA sample preparation kit (Illumina, CA, USA). Multiplex pools were sequenced using MiSeq Reagent Kit v3.0 (Illumina, CA, USA) in paired-end, 250 bp reads. To avoid run-specific variation, case and control SN and FC samples were randomly mixed in each run. All samples were sequenced across 9 runs, average data output per run = 13 Gb (StDev = 1.2 Gb), with an average of 95% > QV30 (StDev = 2.1%). Postrun data, limited to reads with QV ≥ 30, were exported for analysis.

### 2.4 Bioinformatic analysis

Postrun FASTQ files were analyzed using a bioinformatic pipeline. Reads were aligned to the revised Cambridge reference sequence (NC_012920) using BWA v0.7.10 (Li and Durbin, 2009), invoking-mem (Li and Durbin, 2009). Aligned reads were sorted and indexed using Samtools v0.1.18 (Li et al., 2009), duplicate reads were removed using Picard v1.85 (http://broadinstitute.github.io/picard/). Variant calling (including somatic calling) was performed in tandem using VarScan v2.3.8 (Koboldt et al., 2009, Koboldt et al., 2012,) (minimum depth = 1500, supporting reads = 10, base quality ≥ 30, mapping quality ≥ 20, and variant threshold = 1.0%) and LoFreq v0.6.1 (Wilm et al., 2012). Concordance calling between VarScan and LoFreq was >99.5%. Concordant variants were annotated using ANNOVAR v529 (Wang et al., 2010). Perl scripts (available on request) were used to extract base/read quality data and coverage data. MtDNA haplogroup was determined through established algorithms based on existing phylogenetic data (Torroni et al., 1996, van Oven and Kayser, 2009) and using Haplogrep (Kloss-Brandstatter et al., 2011).

### 2.5 Post-bioinformatic QC

In total, 383 samples were sequenced; 174 isolated from the SN (138 cases and 36 controls) and 209 from FC (170 cases and 39 controls). However, to ensure high-quality comparisons, we invoked strict post-bioinformatic quality control (QC). We removed samples if coverage was <99% at a minimal depth of 1500× and removed variants with significant strand biasing (invoked through VarScan). Final analysis was based on 2 cohorts SN = 148 (114 cases, M = 80/F = 35, and 34 controls, M = 17/F = 17) and FC = 155 (125 cases, M = 82/F = 43, and 30 controls, M = 17/F = 15). Heteroplasmic variants are defined as >1% but <99% and homoplasmic variants are defined as >99%. In addition, low-quality variants, present in low-complexity regions, were not included in comparative analysis (Goto et al., 2011).

NGS revealed a high level of genetic variability, identifying 727 unique variants in SNpc tissue samples (n = 148) and 788 unique variants in FC tissue samples (n = 155) (Supplementary Table 1). The proportion of heteroplasmic variants was significantly different between SN (30.4%) and FC (43.8%) (*p* = 0.0001). Although higher in number, the mean comparative macrophage-activating factor (MAF) of heteroplasmic variants was lower in FC compared to SN (MAF = 0.032 and 0.045, respectively) and the difference was larger when restricting the variants to heteroplasmic and nonsynonymous (SN-MAF = 0.035 and FC-MAF = 0.020).

The transition/transversion (Ti/Tv) ratio, a hallmark of selection or random mutation, in human mtDNA has been estimated as 13.75 (Belle et al., 2005). Analysis of sequence data from SN revealed a similar value (Ti/Tv = 13.54, 8.2% transversions); however, FC tissue appeared to have a higher frequency of transversions (Ti/Tv = 9.5, 9.5% transversions) although not significantly (*p* = 7.5 × 10^−2^).

Coding region heteroplasmy (defined as >1%/<99%) was typically low, predominantly <10% in either SN or FC from both cases and controls (Supplementary Fig. 1); although variants >10% heteroplasmy appeared overrepresented in cases, this trend was not significant (SN-cases = 17/270 versus controls = 1/47, *p* = 4.9 × 10^−1^ and FC-cases = 7/223 versus controls = 1/32, *p* = 1.00).

Analysis of total *DLOOP*, *rRNA*, and *tRNA* heteroplasmic variation showed a comparatively higher frequency of heteroplasmic variation than coding regions, but showed no significant differences between cases and controls in either SN (*DLOOP*-P = 6.2 × 10^−1^, *rRNA*-P = 1.0 × 10^−1^, and *tRNA*-P = 2.8 × 10^−1^) or FC (*DLOOP*-P = 5.8 × 10^−1^, *rRNA*-P = 1.1 × 10^−1^, and *tRNA*-P = 4.1 × 10^−1^). Similarly to coding region variants, most rRNA and tRNA variations were low level (<10%) in both SN and FC from both cases and controls; however, DLOOP variation was notably higher (Supplementary Fig. 3).

### 2.6 Somatic mutation analysis

In addition to the original QC, sample pairs were also placed onto the mtDNA phylogeny (www.mitomap). Pairs which did not have concordant mtDNA haplogroups (n = 8) in addition to samples that failed previous coverage-based QC (n = 9) were removed from subsequent analysis leaving a final cohort of 103 individuals (84 cases, and 19 controls).

Using FC tissue as ancestral mtDNA reference sequence and SNpc as the vulnerable, affected, tissue allowed us to identify: “de-novo variants” (present in SNpc, but not FC); “somatic losses” (present in FC, but not SNpc) and “heteroplasmic shifts.” In addition, “positive-heteroplasmic shift” indicates a heteroplasmy increase from FC to SNpc (ΔhetFC < SNpc) and “negative-heteroplasmic shift” indicates heteroplasmy decrease from FC to SNpc (ΔhetFC > SNpc).

Somatic analysis revealed a high level of genetic variability, identifying 1305 differential variants in 103 samples (84 cases and 19 controls). Five hundred fifty-eight were de novo to SNpc, 315 were lost in SNpc, 122 showed an increase in heteroplasmy in SNpc (ΔhetFC < SNpc), and 310 showed a decrease in heteroplasmy in SNpc (ΔhetFC > SNpc; Supplementary Table 2).

### 2.7 Codon bias analysis

Whole Human mtDNA genome data, n = 18,114 sequences, were downloaded from the National Centre for Biotechnology Information Nucleotide database (http://www.ncbi.nlm.nih.gov/nuccore/), using the keyword phrase “Homo [Organism] AND gene_in_mitochondrion[PROP] AND 14000:19,000[SLEN] NOT pseudogene[All Fields].” Sequences with pathogenic mtDNA variants (available at www.mitomap.org) were removed (n = 458 sequences), non-*Homo sapien* sequences were removed (n = 7). Similar to genotype QC, non-European mtDNA sequences (defined with m.8701A, m.8540T, and 10873T) were also removed (n = 7051). Finally, truncated mtDNA sequences (<16,500 bp) were removed (n = 663) leaving a final data set of n = 9935 sequences. The sequence data set was aligned using MUSCLE (Edgar, 2004), analyzed using Haplogrep (Kloss-Brandstatter et al., 2011, van Oven and Kayser, 2009) and subsequently filtered to match the Major European haplogroups (H, V, J, T, U, K, W, X, I, R, and N) leaving a final sample cohort of n = 7729 sequences harboring 2873 variants. Codon usage was calculated as the number of synonymous and nonsynonymous variants per sequence in all 148 SNpc samples, 155 FC samples, and 7729 human mtDNA sequences and is represented as a percentage of the total number of variants per group.

### 2.8 Statistical analysis

Variant counts and heteroplasmy mutation burden were quantified and compared using Linux command line scripts (available on request). Statistical comparisons were performed in SPSS (v15), using data appropriate measurements detailed where appropriate. All group-based test probabilities, that is, loci-specific mutational burden testing in each tissue, were corrected by Bonferroni to correct for multiple significance testing.

## 3 Results

Following strict QC, we compared the frequency of mtDNA variation between PD cases and controls in the largest study of mtDNA variation to date.

**Fig. 1:**
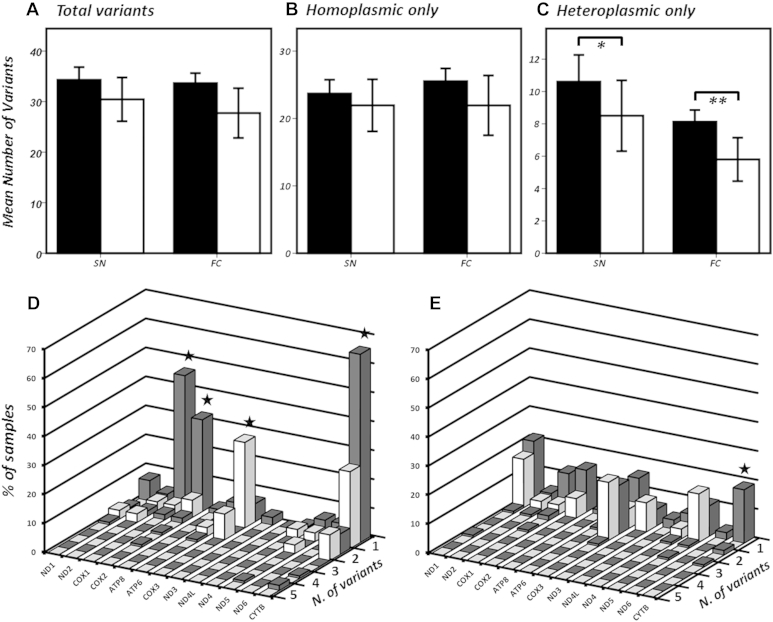
The upper panel shows the comparative mean number of (A) total variants, (B) homoplasmic variants, and (C) heteroplasmic variants stratified by status and tissue origin; where PD = shaded boxes and controls = unshaded boxes. Statistical comparison by Mann-Whitney confirmed a higher heteroplasmic mutational burden in PD patients in both SN and FC (*p = 0.012 and **p = 0.005). The lower panel shows further stratification of heteroplasmic variant burden, limiting to nonsynonomous variants in SN (D) and FC (E) only; where PD = shaded boxes and controls = unshaded boxes. Statistical comparison by Mann-Whitney confirmed a significant (P ≤ 0.0001, starred) overrepresentation of heteroplasmic nonsynonymous variants in PD in *MTCOX1*, *MTCOX2* in SN, and *MTCYTB* in both SN and FC (error bars indicate 95% confidence intervals). Abbreviations: SN = substantia nigra; FC = frontal cortex; PD, Parkinson's disease; COX, cytochrome c oxidase; ATP, adenosine triphosphate.

Stratifying variants by tissue- and variant-type identified a significant difference in the mean heteroplasmic variant burden between cases and controls in both SNpc and FC (*p* = 1.2 × 10^−2^ and *p* = 5.0 × 10^−3^, respectively; Fig 1C). Limiting to nonsynonomous heteroplasmic variation and further stratifying by mtDNA locus revealed a significant overrepresentation of PD cases harboring *MTCOX1*, *MTCOX2*, and *MTCYTB* variants in SNpc and *MTCYTB* variants in FC tissue (Fig 1D and E). These variants were typically low level (predominantly <10%) in both SNpc and FC of PD cases and controls (Supplementary Fig. 1 and Supplementary Table 3); however, assessment of predicted functionality through established pathogenicity scoring (Pereira et al., 2011) revealed significantly higher scores when comparing “total mtDNA variation” (*p* = 1.0 × 10^−2^), *MTCOX1* (*p* = 1.0 × 10^−4^), *MTCOX2* (*p* = 2.0 × 10^−3^), and *MTCYTB* (*p* = 1.0 × 10^−4^) in SNpc tissue between PD cases and controls (Supplementary Fig. 2) an indication that these heteroplasmic variant burdens are potentially detrimental.

Heteroplasmic variation in *MTCOX3* appears overrepresented in control SNpc (Fig. 1D) indicating either a, “protective effect” or more likely a cellular intolerance to further *MTCOX3* mutation in PD samples harboring already increased *MTCOX* variation, a hypothesis supported by observations in Alzheimer's disease (Lu et al., 2010).

Unsurprisingly, heteroplasmic variation in the *DLOOP* accounted for a large proportion of differential variation in both cases and controls, a phenomenon reported in similar studies (Supplementary Fig. 3) (Williams et al., 2013), and *MTATP8*, *MTND4L*, and *MTDN4* appear well-conserved in both PD cases and controls, an indication of mutational intolerance in these particular subunits and again similar to published data. However, we found no significant difference in the distributions of displacement loop, rRNA, or tRNA variants in either SNpc or FC when comparing PD cases to controls.

With previous studies linking PD to mtDNA haplogroup (Hudson et al., 2013, Hudson et al., 2014), we correlated heteroplasmic variant frequency with background mtDNA sequence, but failed to identify a link when analyzing either PD cases or controls in either SNpc or FC indicating no-predisposing link between mutation formation and mtDNA sequence. In addition, and contrary to other studies (Simon et al., 2004), we found no significant correlation to heteroplasmy level or frequencies to either age (Supplementary Fig. 4), which may be cohort selective (i.e., the age range is limited) and limited by the low levels of heteroplasmy we identified, but is also supportive of a recent hypothesis suggesting that heteroplasmic variation formation and expansion primarily occurs in the first half of life (Greaves et al., 2014). Restricting the analysis to PD cases and correlating heteroplasmic variant frequencies to age of onset failed to identify a significant association (Supplementary Fig. 4).

Despite a high number of mtDNA variants, we were unable to identify primary pathogenic mtDNA variants in either SNpc or FC, identifying only alleles putatively associated with several complex traits (Supplementary Table 1). We did identify a single SNpc case harboring the MELAS variant m.3243A>G although this was at a subclinical level (1.02%) and is likely a chance incidental finding.

Limiting to samples where both SNpc and FC tissue was available (cases = 97 and controls = 23) allowed the assessment somatic mtDNA variation. Using FC tissue as ancestral mtDNA reference sequence and SNpc as the vulnerable affected tissue allowed us to identify 4 potential variant classes: (1) de-novo variants (present in SNpc, but not FC); (2) somatic losses (present in FC, but not SNpc); (3) negative-heteroplasmic shifts (a heteroplasmy decrease between FC to SNpc or ΔhetFC > SNpc); and (4) positive-heteroplasmic-shifts (a heteroplasmy increase from FC to SNpc or ΔhetFC < SNpc).

**Fig. 2:**
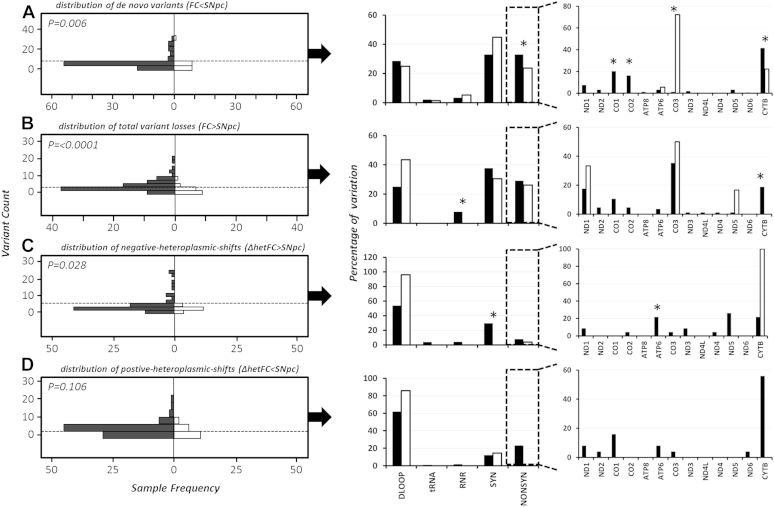
The left panel shows the comparative distribution of (A) de novo SNpc variants; (B) variants lost in SNpc compared to FC; (C) negative-heteroplasmic shifts (ΔhetFC > SNpc); and (D) positive-heteroplasmic shifts (ΔhetFC < SNpc) between PD cases (shaded) and controls (unshaded) using Mann-Whitney nonparametric testing. The middle panel shows the percentage breakdown by variant type, and the right panels shows the mtDNA locus distribution of identified heteroplasmic nonsynonymous variants, where * Pearson's chi-squared P ≤ 0.05 when comparing PD cases and controls. Abbreviations: SNpc, substantia nigra pars compacta; FC, frontal cortex; PD, Parkinson's disease; mtDNA, mitochondrial DNA; ATP, adenosine triphosphate.

Post-QC analysis revealed a significantly increased frequency of de novo mtDNA variants in PD cases compared to controls (*p* = 5.9 × 10^−3^). This variation appears predominantly, in line with our previous data, as nonsynonomous variation in *MTCOX1*, *MTCOX2*, and *MTCYTB* (Fig. 2).

Interestingly, further analysis revealed a significant over representation of somatic-losses and negative-heteroplasmic shifts in SNpc when comparing PD cases to controls (*p* = 1.9 × 10^−5^ and 2.8 × 10^−2^, respectively; Fig. 2), indicating there is strong purifying selection in the SNpc of PD cases, as variants are either removed or heteroplasmy is lowered. Closer inspection of the variant types reveals that PD cases are removing rRNA and synonomous, variants at higher rates than controls; with nonsynonomous variation in *MTATP6* and *MTCTYB* significantly affected (Fig. 2).

Analysis of total positive-heteroplasmic shifts failed to show a significant difference between PD cases and controls (*p* = 1.1 × 10^−1^); however, subsequent stratification by variant type revealed that PD cases have increased heteroplasmy levels in nonsynonomous variants, predominantly in *MTCYTB* (Fig. 2).

## 4 Discussion

The age-related accumulation of heteroplasmic variation is a cardinal component of the biology of aging (Larsson, 2010), yet, the differential spectra of heteroplasmy in human brain tissue and the consequences for age-related disease have not been fully described.

Our data show that increased heteroplasmic mtDNA variation, particularly in *MTCOX1*, *MTCOX2*, and *MTCYTB*, is a hallmark of postmortem PD tissue; genetically supporting early observations that cytochrome c oxidase (COX, complex-IV) activity is an important component in PD (Gu et al., 1998, Kraytsberg et al., 2006, Rana et al., 2000, Shinde and Pasupathy, 2006) and indicating that heteroplasmic variation likely contributes to the decreased level of COX subunit expression seen in the brains of neurodegenerative patients (Aksenov et al., 1999). Contrary to this hypothesis, the variants we identified were typically low level (predominantly <10%) in both SNpc and FC of PD cases and controls raising the possibility that either: (1) these variants are benign and unlikely to result in a biochemical deficit or (2) they are deleterious and have contributed to neuronal cell death in cells where they have clonally expanded. The latter seems more likely, given the significantly different predicted pathogenicity seen between cases and controls; however, functional experiments will be needed to fully investigate this.

Similar to previous studies (Stewart et al., 2008), we found a decrease in the number of first and second codon variants compared to third codon variants in both SNpc and FC when analyzed collectively (Supplementary Fig. 5), suggestive of purifying selection. However, when compared to population controls, both SNpc and FC showed a significant overrepresentation of nonsynonymous codon 1 variants (SNpc = 0.04 and FC = 0.04) compared to the human sequences, further suggesting increased overall mutagenesis in the brain.

Interestingly, given the putative role of complex I in PD and aging (Mizuno et al., 1989, Smigrodzki et al., 2004), we found relatively few MTND* variants. One, m.12680 (*MTND5*) has been previously reported in PD brains (Smigrodzki et al., 2004). This may indicate that in fact complex I variation is not a component of PD or may reflect the lack of remaining SNpc in postmortem, late-stage, PD cases, where pathogenic MTND* variants have already resulted in cell death (Cantuti-Castelvetri et al., 2005).

Somatic mutation analysis suggests that the SNpc of PD cases undergoes strong purifying selection, particularly removing or depleting nonsynonomous heteroplasmic variation when compared to matched FC tissue. However, our data suggests this is a losing battle, as PD cases also show an increase in the formation of de novo nonsynonomous *MTND1*, *MTND2*, *MTCO2*, *MTCO3*, and *MTCYTB* variants in the SNpc compared to controls.

The identification of variants both lost and gained in SNpc (when compared to FC) appears initially paradoxical, although it is likely that this is either a product of end-stage PD; where cell death has removed the causative variants leaving only the healthiest cells, or it is a result of the “viscous cycle” of mutation formation seen during the increased mitochondrial dysfunction seen in PD (Swerdlow et al., 1996). The most likely explanation is a combination of both phenomena; as comparative pathogenicity scores of somatic, nonsynonomous, heteroplasmic variation (Supplementary Fig. 6) and an increased mutational burden in PD SNpc compared to controls, indicate that detrimental mutation formation is superseding mutation loss. In addition, studies have shown that low-level heteroplasmic variants can escape the murine inheritance bottle-neck and may impair brain development. The inclusion of further tissue samples, such as tissue from nonectodermal lineages could be used to investigate the origins of somatic mutation further, although this tissue is not always readily available from brain tissue banks.

Finally, we also recognize some study limitations. The availability of viable, nonpathological control, tissue limits the power of the comparisons we can make. We have mitigated this in part, studying a large cohort of PD cases and investigating the effects of somatic mtDNA variation; however, replication of these findings in an independent cohort is warranted.

### 4.1 Conclusion

Here, using established NGS techniques, we show that heteroplasmic and somatic mtDNA variation is a hallmark of PD. Our data indicate that the brain is in a state of mutational flux, both gaining and losing mtDNA variation over time, but more importantly, our study confirms the important role of age-related mtDNA point mutation in the etiology of PD, moreover, by analyzing 2 distinct brain regions, we are able to show that PD patient brains are more vulnerable to mtDNA mutation overall. However, if the role of mtDNA mutation is to be fully understood, replication of these findings in an unrelated cohort and further investigation of the functional role of this variation is recommended.

## Disclosure statement

The authors declare that they have no competing financial interests.
