# Supplementary material for: Somatic mtDNA variation is an important component of Parkinson's disease
Source: Neurobiol Aging. 2016 Feb;38:217.e1–6. doi: 10.1016/j.neurobiolaging.2015.10.036 (PMC4759607; doi:10.1016/j.neurobiolaging.2015.10.036)
Supplement: Supplementary Figs. 1–6 [file mmc1.docx]

***Supplementary Figure legends***

**Supplementary Fig. 1.** Distribution of coding heteroplasmic variants in PD cases (shaded) and controls (unshaded) in SNpc **(a)** and FC **(b)**, showing percentage of overall variant counts per gene per heteroplasmy. The graphs indicate that the overall levels of heteroplasmy in the brain are low, although cases do exhibit slightly higher levels (Supporting raw count data in Supplementary Table 3).

**Supplementary Fig. 2.** Comparative mtDNA pathogenicity scores for heteroplasmic non-synonomous variation in PD cases (shaded) and controls (unshaded) in both SNpc and FC, showing ‘Total’ mtDNA (P=1.0x10^-2^), *MTCOX1* (P=1.0x10^-4^), *MTCOX2* (P=2.0x10^-3^) and *MTCYTB* (P=1.0x10^-4^).

**Supplementary Fig. 3. (a)** Distribution of non-protein coding (*DLOOP*, *tRNA* and *rRNA*) heteroplasmic variants in PD cases (shaded) and controls (unshaded) in SNpc (left) and FC (right) and **(b)** distribution of non-coding heteroplasmic variants in PD cases (shaded) and controls (unshaded) in SNpc (left) and FC (right), showing percentage of overall variant counts per locus type, per heteroplasmy (Supporting raw count data in Supplementary Table 3).

**Supplementary Fig. 4.** Correlation of total heteroplasmic mutation burden to age, stratifying PD cases (shaded) and controls (unshaded) in both SNpc and FC (upper panels) failed to find a significant association. Similarly, correlation of heteroplasmic mutation burden to age of onset, stratified by tissue failed to find an association (lower panels).

**Supplementary Fig. 5.** Relative codon usage, stratified by synonymous (unshaded) and non-synonymous (shaded) variants and status (where PD= PD cases and C=controls) detected in substantia nigra pars compacta (SNpc, n=148), frontal cortex (FC, n=155) and a representative human mtDNA population control (n=7739), showing an increase in the frequency of non-synonomous codon 1 and 2 variants compared to human control sequences.

**Supplementary Fig. 6.** Significantly different (P=2.9x10^-3^) comparative mean pathogenicity score **(a)** for heteroplasmic, non-synonymous, somatic mtDNA variation (SNpc>FC) between PD cases (shaded) and controls (unshaded). Additionally, PD cases showed a significantly higher individual pathogenicity burden **(b)** compared to controls (P=4.0x10^-3^).

**Supplementary Fig. 1.**

**
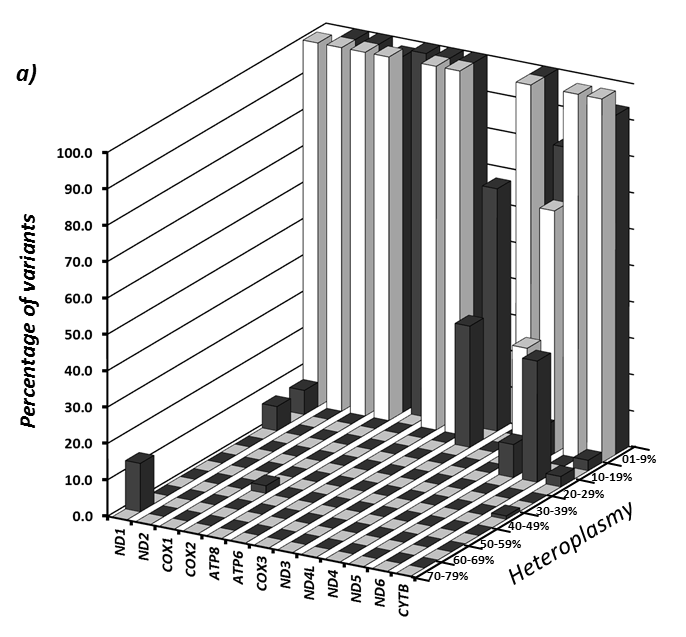
**

**
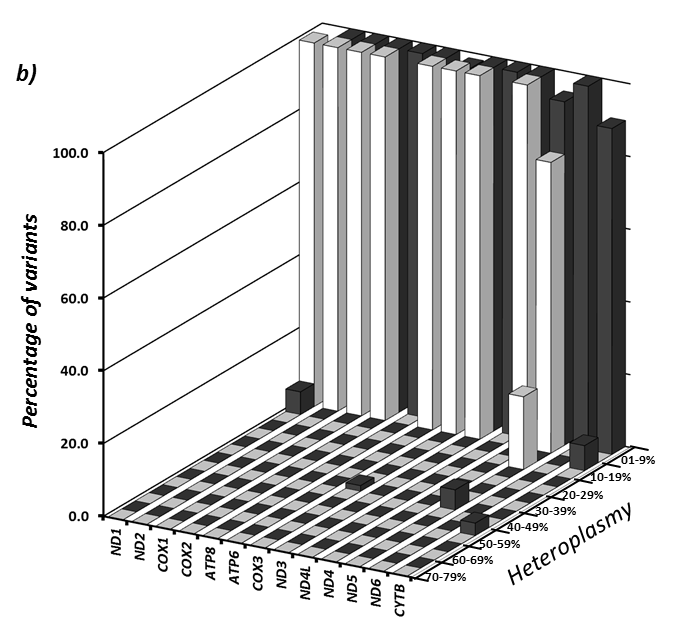
**

**Supplementary Fig. 2.**

***
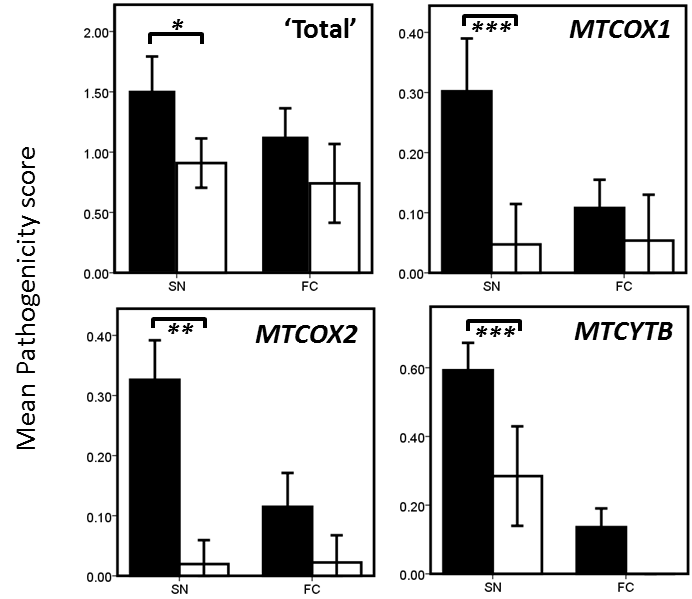
***

**Supplementary Fig. 3.**

**
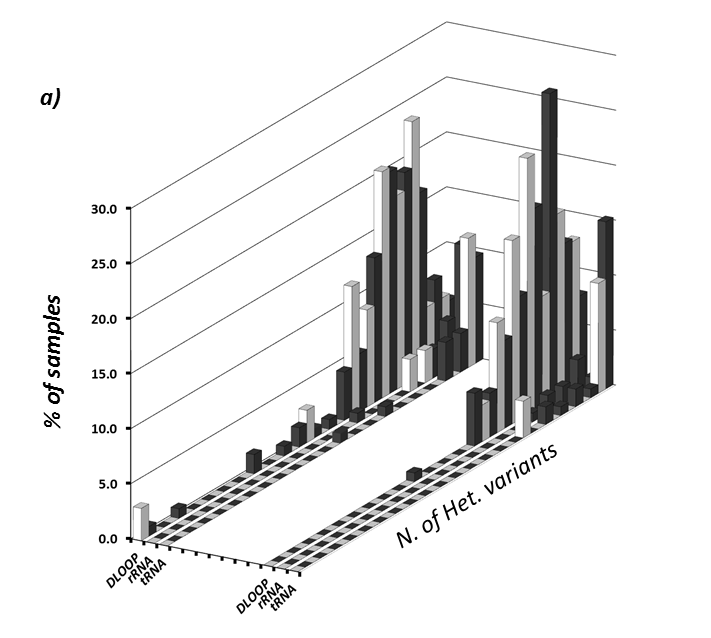
**

**
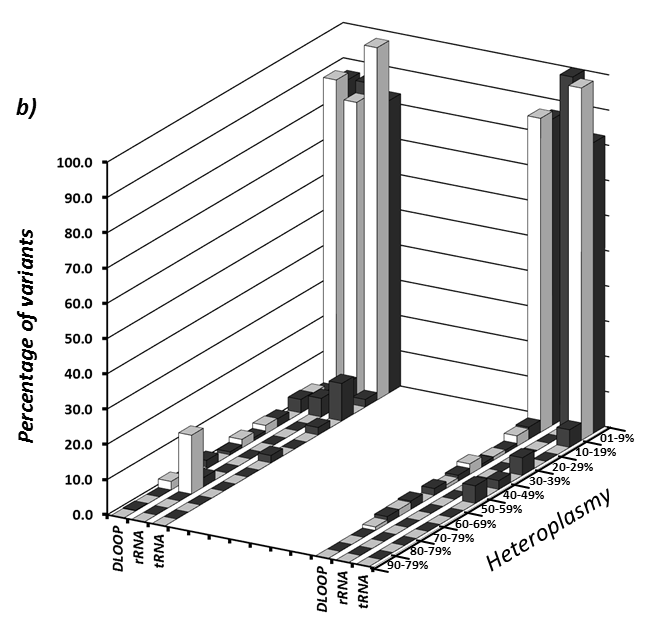
**

**Supplementary Fig. 4.**

**
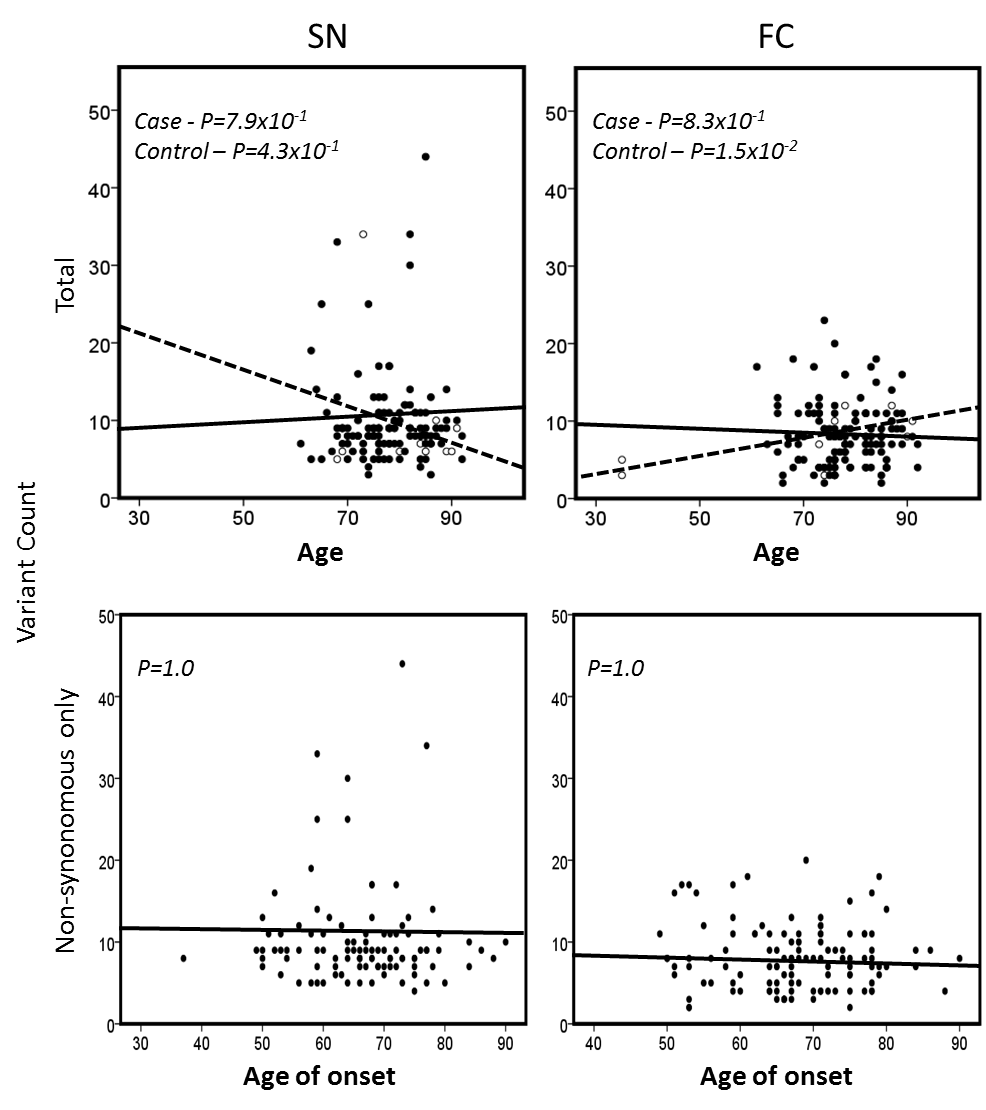
**

**Supplementary Fig. 5.**

**
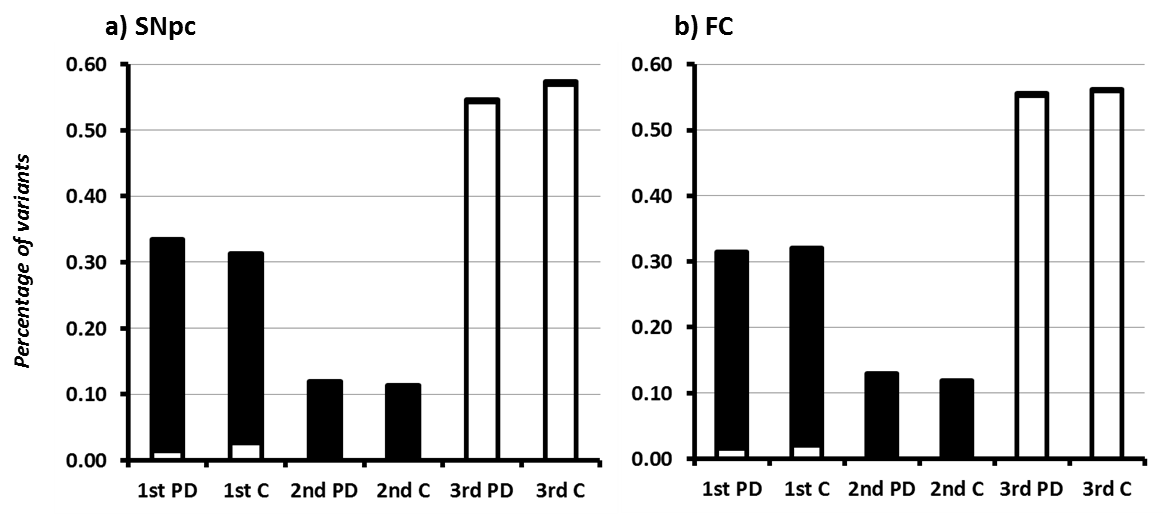
**

**Supplementary Fig. 6.**

**
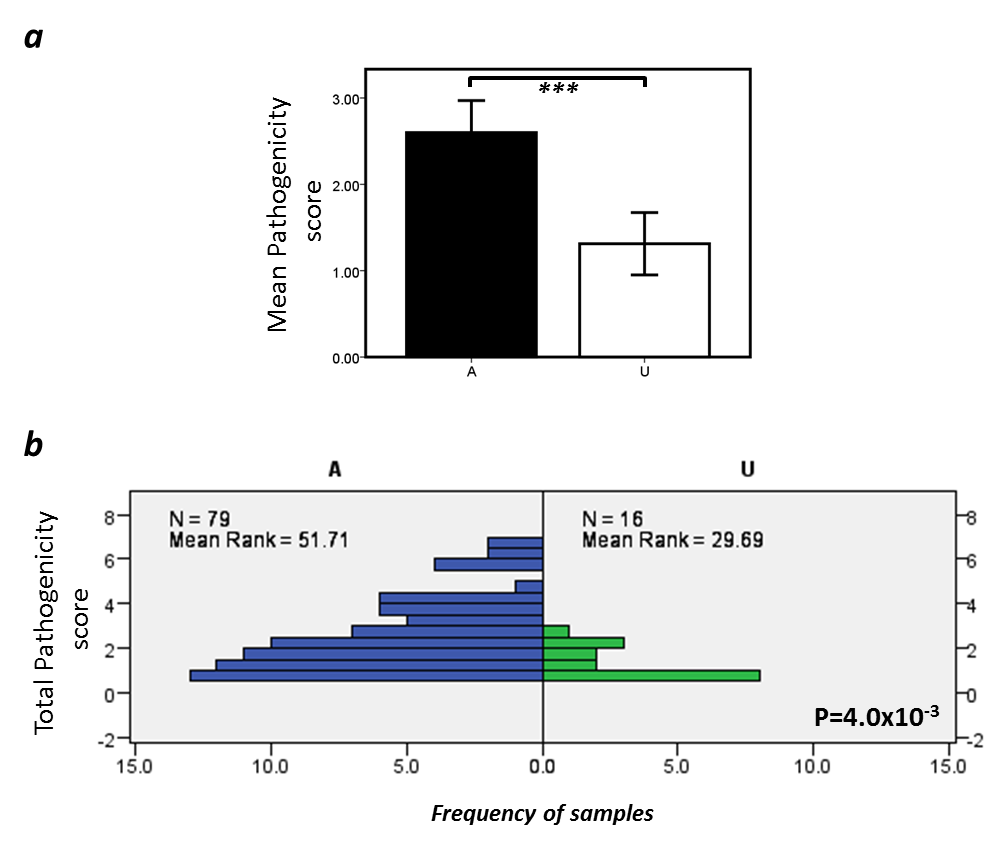
**
